# Former Buruli Ulcer Patients’ Experiences and Wishes May Serve as a Guide to Further Improve Buruli Ulcer Management

**DOI:** 10.1371/journal.pntd.0005261

**Published:** 2016-12-29

**Authors:** Anita Velink, Rebecca J. Woolley, Richard O. Phillips, Kabiru M. Abass, Tjip S. van der Werf, Emmanuel Agumah, Janine de Zeeuw, Sandor Klis, Ymkje Stienstra

**Affiliations:** 1 University of Groningen, University Medical Center Groningen, Groningen, The Netherlands; 2 Kwame Nkrumah University of Science and Technology, School of Medical Sciences, Department of Medicine, Kumasi, Ghana; 3 Agogo Presbyterian Hospital, Agogo, Ghana; 4 University of Groningen, University Medical Center Groningen, Department of Internal Medicine/Infectious Diseases, Groningen, The Netherlands; 5 President and founder of Buruli ulcer Victims Aid (BUVA) foundation, Kumasi, Ghana; 6 University of Groningen, Institute for Medical Education, Learning Community Global Health, Groningen, The Netherlands; University of California San Diego School of Medicine, UNITED STATES

## Abstract

**Background:**

Buruli ulcer (BU), caused by *Mycobacterium ulcerans*, is a neglected tropical disease frequently leading to permanent disabilities. The ulcers are treated with rifampicin and streptomycin, wound care and, if necessary surgical intervention. Professionals have exclusively shaped the research agenda concerning management and control, while patients’ perspective on priorities and preferences have not explicitly been explored or addressed.

**Methodology/Principal findings:**

To get insight into patient perception of the management and control of Buruli ulcer a mixed methods research design was applied with a questionnaire and focus group discussions among former BU patients. Data collection was obtained in collaboration with a local team of native speakers in Ghana. A questionnaire was completed by 60 former patients and four focus group discussions were conducted with eight participants per group. Former patients positively evaluated both the effectiveness of the treatment and the financial contribution received for the travel costs to the hospitals. Pain experienced during treatment procedures, in particular wound care and the streptomycin injections, and the side-effects of the treatment were negatively evaluated. Former patients considered the development of preventive measures and knowledge on the transmission as priorities. Additionally, former patients asked for improved accessibility of health services, counselling and economic support.

**Conclusions:**

These findings can be used to improve clinical management and to guide the international research agenda.

## Author Summary

Buruli ulcer (BU) is a skin and soft tissue infection possibly leading to deformities and long term consequences with severe impact on patients’ lives. It is one of the neglected tropical diseases. The current research agenda is created solely by health professionals, whereas patients might indicate different priorities. Therefore we conducted qualitative research on former patients’ experience with the BU management and on the priorities for the research agenda as envisaged by patients. We found that the preferences of former patients appeared generally in agreement with the international research agenda. Additionally, they indicated that additional preventive measures, better accessibility to care and pain management were important subjects for them. More counselling and social support in the form of community-based rehabilitation were expected to contribute in dealing with the impact of the disease and to enlarge a public platform for support. For clinical management we found that more information on the disease appears necessary, since former patients indicate they did not know much about the course of treatment and expected side-effects. Involving patients can improve BU management and its research agenda.

## Introduction

Buruli ulcer (BU) is a devastating skin and soft tissue infection prevalent in several tropical and subtropical endemic areas worldwide, with the highest prevalence in West Africa [1,2]. BU is caused by infection with *Mycobacterium ulcerans (M*. *ulcerans)*. It is the third most common mycobacterial disease in immunocompetent persons [3,4]. In early disease stages BU presents as a painless nodule, plaque or oedema. Without treatment these lesions go on to ulcerate presenting with characteristic undermined edges. The lesions are divided in three categories; Category I: a single lesion <5cm in diameter. Category II: a single lesion 5-15cm in diameter. Category III: single lesion >15cm in diameter, multiple lesions, lesions at critical sites such as eye, breasts, genitalia and osteomyelitis [5]. Drug treatment for BU consists of a combination of antibiotics given for an eight weeks period. The current World Health Organization (WHO)-recommended regimen is rifampicin (10 mg/kg once daily, oral tablet) combined with streptomycin (15mg/kg once daily, intramuscular injection). Wound care is performed three times a week to daily depending on the severity of the wound. Almost 30% of patients indicate pain during the wound care procedure [6]. Physiotherapy is required to promote healing of the wounds and to prevent disabilities [7]. Over 90% of the early detected, limited cases (<10cm diameter) can be treated with antibiotics alone and have a good quality of life in long-term follow-up [8,9]. Health seeking behaviour among BU patients is complex [10–12]; If BU patients delay in seeking medical care, they run an increased chance to develop complications [13–15]. Patients who present with larger lesions are more at risk of developing contractures, functional limitations, and social participation restrictions [15,16]. Surgical treatment, including skin grafting may be necessary in patients with complicated lesions or without satisfying response to antimicrobial treatment.

The pathological mechanisms, tissue necrosis, immune suppression as well as the painlessness of the ulcers, are mediated by the toxin mycolactone produced by *M*. *ulcerans* [2,17].

Although *M*. *ulcerans* is believed to be acquired from environmental sources in endemic areas, the exact mode of transmission is currently incompletely understood. Risk factors identified that increase susceptibility for BU include being aged below 15 years or over 49 years, poor hygiene of existing wounds, living close to stagnant water, insect bites, the water sources used and the activities near them [18,19].

The current research priorities as stated by WHO are the mode of transmission, development of methods for early diagnosis, drug treatment and new treatment modalities, vaccines, cultural and social-economic studies and lastly, incidence, prevalence and mapping of BU [20]. Patient’s perspectives on a disease may however lead to different priorities compared to researcher’s priorities [21–23].

This study questions former BU patients about their experiences and the congruent priorities for research and interventions to improve management of BU.

## Methods

### Study area

The study was conducted in two endemic rural areas in Ghana, near Agogo Presbyterian Hospital and Nkawie/Toase Governmental hospital in Agogo and Nkawie respectively. Both hospitals are important BU treatment centers in their areas. In 2015, the hospital in Agogo treated 17% of the new BU patients registered nationally.

### Study design

A mixed method design was used consisting of questionnaires with open-ended and multiple choice questions and focus group discussions. Between June and November 2012 the questionnaires were administered. Thereafter between August and November 2014 focus group discussions were organized in order to further discuss the topics identified, combined with patients’ experiences, perceptions and suggestions for improvements and future research. Four focus group discussions were held; the number of focus group discussions chosen was based on reported evidence that 70–80% of relevant topics were identified after four discussions [24].

### Participants

Former BU patients residing in the catchment area of Agogo and Nkawie were eligible for participation. Participants were at least 16 years old, healed from a small BU lesion (Category I or II) within a year after start of treatment. Community volunteers were instrumental in localising them on their last known address in hospital administration. The questionnaires were administered to 71 participants. These participants in the BURULICO trial between 2006 and 2009 could be retrieved for follow-up visits on long-term consequences as described in Klis et al [25].

The focus group discussions were conducted with eight randomly selected participants per group, selected by purposive sampling based on hospital records. The aim was to obtain a heterogeneous group concerning age, profession and education to encourage the discussion. One female group and one male group discussion were conducted in each area, so in total 16 females and 16 males participated in the discussion. Male and female groups were chosen due to existing gender inequalities in traditional Ghanaian communities [26,27]. Therefore it was expected that participants would be more open and feel more at ease to express their opinions and experiences in homogenous groups [28,29].

### Data collection

Due to a high rate of illiteracy among the participants, all questionnaires were administered orally in their native language. Three trained local hospital staff members administered the questionnaires in a quiet private place.

The focus group discussions were performed by a local team organised according to the principles specified on organising focus group discussions in low-income countries [24]. Preliminary testing, training of the moderator and note taker, and a pilot study with hospital staff were completed before data collection. The participants were given a reimbursement for their transportation and participation when the groups were finished. The recordings of the focus group discussions were then translated and transcribed by the note taker, who supplemented the recordings with his notes made during the meetings. The translation and transcription were checked by a second member of the research team.

### Data analysis

Qualitative analysis was performed on the final English transcripts using open coding and axial coding. During the open coding, the English transcripts were read carefully and codes were assigned, which was done individually by two people (AV and RW). The initial codebook was developed based on the open coding of two interviews. Thereafter, the two separate codebooks were discussed and combined in regular meetings. In case of disagreement other researchers (YS and JDZ) were consulted in order to reach consensus. Throughout data collection the codebook was adapted based on new codes that emerged from the data. After the open coding, the various codes were subjected to a process of axial coding in order to develop categories and identify connections.

### Ethics

The study protocol was approved by the Committee on Human Research, Publication, and Ethics of the Kwame Nkrumah University of Science and Technology and the Komfo Anokye Teaching Hospital, Kumasi (reference number CHRPE/RC/158/14).

Informed consent or assent for the questionnaires and all focus group discussions was explained and obtained and the forms were signed by signature or thumbprint by participants and by parents or legal representatives in participants <18 years.

## Results

### Participant characteristics

All participants approached for the questionnaires were willing to participate. Of the 71 administered questionnaires, 11 were incomplete and 60 were included in analysis.

The majority of these participants were females (N = 42; 70%). The age of the participants ranged between 16 and 77 years, with a median of 23 years (IQR (25–75%) 18–31). All these participants had lesions < 10 cm in diameter.

One of the 33 persons approached for the focus group discussion refused due to work and time-related issues. The age distribution was from 18 till 73. Only 3 former patients in this group had had category 3 lesions.

### Findings of the questionnaires concerning experiences of the former patients

**Fig 1:**
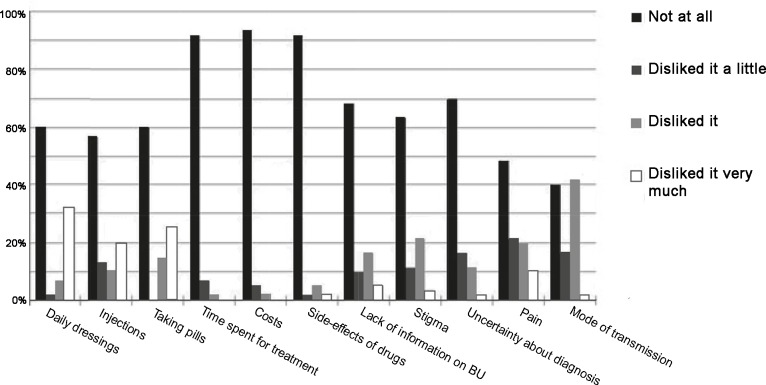
summary of multiple-choice questions analysis–scores represent Likert scales of increasing discomfort with treatment aspects.

The results of the questionnaire on the multiple choice questions exploring which aspects of suffering from BU was important, with a Likert scale reflecting the degree of their dissatisfaction, are shown in Fig 1. 49 former patients (82%) reported not to have disliked the treatment aspects they were interviewed about in the open questions, but in the multiple choice questions, only 4 (6.7%) indicated no dislike for any of the aspects mentioned. More than 50% of the participants disliked the pain they experienced during treatment. Other striking results include that 60% of participants *disliked* the uncertainty about how they contracted the disease. For 22% of these participants, this was their only dislike.

Furthermore, during the open questions, 27 participants(45%) didn’t mention anything. 15 participants (25%) found it bothersome that they ran into trouble with work or school. Being bothered by side-effects, pain, stigma and having to walk a long distance to receive treatment were also mentioned. Moreover, when asked what they disliked, 11 participants (18%) mentioned injections, pills, dressings and punch biopsies. When asked what they liked, a third of the participants reported to appreciate the small incentive they received. The effectiveness of treatment was mentioned as well; 13 participants (22%) enjoyed the way they were welcomed and treated in the hospital. Additionally, singular aspects of treatment were mentioned as well as their effectiveness. We did not explore why these things were considered positive.

### Findings of the focus group discussions concerning experiences and suggestions of the former patients

#### Information received on the disease and treatment

Almost half of the participants stated they were not given enough information. Participants indicated that they did not know where to turn to when they developed symptoms of BU. Most of the participants were directed, either by community volunteers or other people who knew about it, or had had the disease before. A few mentioned that they received previous education and information on BU in the community or that they had heard about the disease from others.

#### Opinions about wound dressings

The opinions on dressings were very unanimous. Dressings were found to be very painful but very effective. *‘‘It was very effective*, *because I realized it was getting better and better each day*.*”* A lot of examples and reasons for pain during changing of dressings were mentioned. *‘‘The dressing of the wound is very painful*, *especially when they are removing the previous gauze on the wound”* No option for improvement or changes to reduce the pain during dressings were given.

#### Experiences of treatment

The experiences of treatment were very broad. Considering the effectivity, the participants did not mind the eight weeks duration of the treatment in general. Women compared to men mentioned more problems and negative experiences with going to the health centre. *‘‘I was walking long distance everyday”*. In addition, many participants mentioned severe side-effects and problems with the injections and/or tablets. The injection was mainly found to be painful, additionally some were scared of the needles or mentioned other aspects. *‘‘I also preferred the tablet over the injection*, *because after receiving the injection*, *I sometimes bled after the injection and before I reached home*, *my uniform will be all soaked with blood*. *‘‘*

Side-effects mentioned ranged from very scary urine changes, rashes, dizziness to hearing problems and loss of appetite. *‘‘after taken the drug your urine would change colour to red”* Most participants preferred the tablet. Only two participants reported to have problems with the tablets. *‘For me*, *swallowing of the tablet or capsule was a problem to me*, *so I have to take it with food or put it into rice ball before I can swallow it*.*”*

#### Physiotherapy and POD

The discussion on the topics of physiotherapy and the prevention of disability (POD) was mainly focused on the fact that though painful it was helpful to have physiotherapy when they had the disease. *‘‘…and it was very painful*, *I always thought he was very cruel*, *till it got healed without limitation that I realised that he was rather helping me*.*”* Suggestions for improving physiotherapy and POD focused on teaching the patients how to do it themselves and training members of the community or family to assist. *‘‘I suggest there should be certain people who would be from the community that would assist the victims in doing exercise‘‘*.

#### Counselling

Overall, a large majority (71%) of the participants’ remarks indicated the disease somehow socially and/or economically affected them. They either were unable to work or got financial or family problems or they were stigmatized by their fellow villagers. So when discussing the need for counselling, the participants in all groups agreed that more counselling is necessary for various reasons. *‘‘I strongly support there is the need for counselling*, *because when one gets the disease*, *you will realised family and friends begin to keep themselves away from you and that alone make you feel sad*.*”*

#### Vaccination

When asked about the possibility of getting a vaccination to prevent the disease, all participants fully agreed that that would be a very good idea and they fully supported this idea. *‘‘I truly support participant nr*. *7’s suggestion*, *because it is said that 'prevention is better than cure'*.*” ‘‘I would be grateful to the Buruli ulcer team if they can get a vaccine for the disease”*

#### Most important for future research

In the concluding question some focus went again to the topic of vaccination, since that would prevent the disease. Another important topic mentioned was education on the disease and the importance of early recognition. Also a few mentioned that they thought research should focus on how the disease is transmitted, mainly because then the disease might be preventable. *‘‘Again my concern is that we do not know how one gets this disease*, *whether it is by*, *on or from the river*. *We cannot exactly feel how we get it*, *but is just a presumption that we get it from the river and for that matter I will urge the government to carry out a research on our river bodies and also get medicine that can eradicate this disease*.*”* Changes in medication were also wanted, especially eliminating the injection from the treatment regime and just supporting the medication and its availability in general. There were also requests for more support for the victims of BU. *‘‘… because people of this village called victims by the names “bankruptcy wound”*.*”* Support for the doctors and nurses were also mentioned as well as increasing the number of health workers. The accessibility of the health services were also discussed, since the clinics were sometimes too far away to walk and not easily accessible by transport. *‘‘Other aspect that I think it should be improved*, *should be availability of transportation for victims to easily reach their various hospital and clinic to seek treatment*. *My saying this*, *because most of us find it very difficult to get access to vehicle to reach the hospital*. *‘‘* And lastly it was also mentioned that the government could try to help out more in the rural areas and could try to protect people from getting the disease. *‘‘I would plead to the government to reduce prices of farm protective clothing and shoes*, *so that farmers can afford them*. *Because most of us work on our farms without these items and it has become very difficult for us to protect ourselves from this disease*, *eventually coming home with this disease*. *Again if the government can do their best to improve our sanitation situation in the village*, *because I think dirt also contributes to the onset of the disease*.*”*

## Discussion

The study aimed to describe the experiences and needs of BU patients, to determine if former BU patients have the same priorities as the current WHO research agenda and if this information might benefit effective management and treatment of BU.

Experiences were elaborately described and very diverse. Several negative aspects of BU management were identified. Many negative aspects were associated with pain, especially during dressings, injections, and POD activities, which confirmed recent reports on pain being common in BU [6, 30].

During the focus group discussions, in both areas, as well as between males and females, hardly any discrepancies could be detected. Women were a little more elaborate on the social consequences and the difficulties with accessing health care while men were slightly more focused on preventative methods, like protective clothing. Negative experiences of treatment were mostly associated with pain during dressings, physiotherapy or injections. This corresponds with the findings of de Zeeuw et al. (2015) that pain management in BU needs more attention [30].

In addition, preventive measures and knowledge on transmission were frequently mentioned as points for improvement. Community based rehabilitation as suggested by the world report on disability would be appreciated by and be beneficial to patients. Tuakli Wosorna and Haig (2014) described that due to bidirectional social stigma these have not been very helpful so far in Ghana [31]. Therefore it is important to encourage the empowerment and acceptation of former patients. Furthermore, a high demand for support to deal with the economic and social burden and need for counselling emerged from the focus group discussions.

The findings of the questionnaires showed that many participants appreciate the small incentive they were given to come to the hospital. This confirms data from Ahorlu et al. (2013), who found that social interventions, like free transportation and breakfast were beneficial for early case detection and treatment, thus preventing further complications [32].

A noteworthy result is that former patients felt that they lacked information about the treatment options and why the treatment would be carried out in a certain way. Uncertainty about where to seek treatment and lack of knowledge about BU before they were diagnosed were also mentioned. Indeed, early diagnosis and treatment is associated with better outcome [13,14]. Several people in the focus group discussions mentioned that they were scared because their urine changed colour during treatment, whilst this is invariably the case in individuals taking rifampicin [33].

### Limitations study

The questionnaires helped to identify topics for the focus groups discussion but were administrated two years earlier than the focus groups discussions. During this period patients’ experiences and opinions potentially could have changed. As BU treatment and management did not change in these two years, we do not expect any impact on our results. Furthermore, only former patients with small lesions (Category I or II) were included in the study. Former patients or patients with larger lesions might have indicated other priorities. Not all participants had BU in the same period, it might introduce recall bias. However, this recall problem could not be detected during the study and it was not mentioned by participants.

In the overall results there was a lack of argumentation and support of statements made by our study participants. This may be because participants are not aware of other possibilities to do certain treatment aspects differently. Additionally, Ghana is known as a hierarchical culture that is quite collectivist; ‘The doctor knows best’ [34]. This may have led participants to not critically appraise their doctors and the treatment they received. Another reason might be that the participants, though assured they should say anything they had on their mind, nonetheless did not feel free to express all of their thoughts.

The participants of the focus group discussions easily reached agreement with limited discussions as a result, despite all focus groups consisting of a heterogeneous group and the facilitator stimulating discussion. Possible explanations can be that the former patients had similar experiences concerning treatment or that a negative group dynamic occurred. However, during the discussion all were encouraged equally to express their opinion. During the discussion equal opportunities for expression were monitored and all participants were involved in order to minimize effects of group dynamics. We therefore conclude that limitations due to negative group dynamics were minimal.

Members of the local BU team were working on the study; this might have affected the participants’ sense of freedom of expression, though all members not vital for the discussion were asked to leave the area where the groups met. To promote participants to express their opinions a former BU patient was designated as moderator. By inviting a former BU patient, and not a staff member, we aimed that participants would feel more comfortable during the discussion, knowing that the moderator could identify with them, and encourage the discussion effectively.

## Conclusion

The preferences and priorities of BU patients appeared generally in agreement with the international research agenda. Additional preventive measures, accessibility and pain were mentioned as important subjects. More counselling would benefit some patients, since the disease may have a huge impact on life, economically as well as socially. Community based rehabilitation was suggested, thus enlarging the public support for the disease as well as benefitting the patients directly. In addition, for clinical purposes, more explanation on the course of treatment and expected side effects appears necessary, since a lack of knowledge on these aspects was indicated. Doctors are limited in their time to explain issues extensively, but giving more elaborate information on the management of the disease and possible side-effects appears to be useful to enhance compliance to treatment.

This information could aid in creating a patient based BU research agenda in which patient empowerment in neglected tropical diseases may improve healthcare.

## Supporting Information

S1 Checklist “S1 Checklist. STROBE checklist.”
(PDF)
